# Prenatal Preview: Early Bisphenol A Exposure May Spawn Late-Life Reproductive Problems

**DOI:** 10.1289/ehp.117-a256a

**Published:** 2009-06

**Authors:** Angela Spivey

Scientists increasingly find that exposure to toxicants during critical periods of pre- and perinatal development can have long-lasting effects that increase the risk of reduced fertility, reproductive tumors, and breast cancer later in life. Some animal studies have suggested that very low doses of bisphenol A (BPA) in a range relevant to human exposures can cause abnormalities in the uterus, vagina, and ovary when those exposures occur during early development. To date, however, evidence for a carcinogenic effect of low-dose BPA on the female reproductive tract has been lacking; studies examining carcinogenic susceptibility to BPA have focused only on the mammary and prostate glands. Now a report from researchers at the NIEHS indicates that low prenatal doses of BPA in mice may cause potentially carcinogenic alterations in female reproductive tissues **[*EHP*** **117:879–885; Newbold et al.]**.

Widespread human exposure to BPA is a cause for concern because of the compound’s chemical similarity to diethylstilbestrol (DES), an antinausea drug that caused harmful reproductive effects in women whose mothers took it during pregnancy. This study used an experimental animal model (the CD-1 mouse) that has been useful in studying the effects of prenatal exposure to DES.

On gestational days 9–16, pregnant mice were injected with relatively low dosages of BPA—0.1, 1, 10, 100, or 1,000 μg/kg/day, which are considered by the authors and other researchers to be within an environmentally relevant range. When the dams’ offspring reached late adulthood (16–18 months), their reproductive tissues were evaluated.

Some of the BPA-exposed offspring developed both benign and malignant lesions in late adulthood. In the pups that received 1 μg/kg/day, the incidence of benign ovarian cysts was 67%—similar to what the same team had previously observed for neonatal exposure to BPA. In addition, more severe ovarian lesions were found in the groups that received 10, 100, or 1,000 μg/kg/day but not in control animals. The occurrence of progressive proliferative lesions of the oviduct seemed to increase following BPA exposure, similar to effects seen in previous studies following prenatal exposure to DES. Because DES appears to delay the expression of genes that guide the development of the reproductive tract, the authors suggest that molecular “misprogramming” is the most likely reason for both DES- and BPA-induced lesions of the oviduct.

Malignant changes in the uterus also were found in exposed mice, although the incidence of those lesions was not statistically different from that seen in controls. In addition, more severe pathologies were observed in exposed animals but not in controls. These included atypical hyperplasia and stromal polyps of the uterus, sarcoma of the uterine cervix, and mammary adenocarcinoma.

According to the authors, this study is the first to find both benign and malignant lesions in reproductive tissues of senescent female mice exposed prenatally to BPA over a wide dosage range thought to be relevant to human exposure. The study adds to the growing body of literature showing adverse effects following developmental exposure to low doses of BPA and suggests that exposure during critical periods of fetal development may result in adverse reproductive and carcinogenic changes over the long term.

